# Influence of COVID-19 on Clinical Outcomes of *Clostridioides difficile* Infection

**DOI:** 10.3390/antibiotics15080806

**Published:** 2026-08-18

**Authors:** Martin Novotny, Daniela Javorska, Annamaria Toporova, Nicolas Kardos, Katarina Curova

**Affiliations:** 1 Department of Infectology and Travel Medicine, Faculty of Medicine, Pavol Jozef Šafárik University in Košice, Rastislavova 43, 04190 Kosice, Slovakia; martin.novotny@unlp.sk (M.N.); daniela.javorska@student.upjs.sk (D.J.); 2 Department of Medical and Clinical Microbiology, Faculty of Medicine, Pavol Jozef Šafárik University in Košice, Trieda SNP 1, 04011 Kosice, Slovakia; annamaria.toporova@upjs.sk; 3 Department of Paediatrics and Adolescent Medicine, Faculty of Medicine, Pavol Jozef Šafárik University in Košice, Trieda SNP 1, 04011 Kosice, Slovakia; nicolas.kardos@upjs.sk

**Keywords:** *Clostridioides difficile*, COVID-19, *Clostridioides difficile* infection, CDI

## Abstract

**Backgroud/Objectives:** *Clostridioides difficile* infection (CDI) remains a major problem for healthcare facilities. The COVID-19 pandemic was associated with increased hospitalizations, frequent antibiotic use, and changes in infection prevention and control regulations, which may have influenced the epidemiological and clinical characteristics of CDI. The aim of this study was to evaluate whether a history of COVID-19 was associated with clinical characteristics, treatment exposure, recurrence, ribotype distribution, and mortality in patients with CDI. **Methods:** This retrospective study included patients diagnosed with CDI during the period 2020–2022 at the Louis Pasteur University Hospital in Košice, Slovakia. We analyzed 324 patients, of whom 172 (53.1%) had no history of COVID-19 and 144 (44.4%) had previous or concurrent COVID-19, defined as documented infection within three months before or at the time of CDI diagnosis. Eight patients (2.5%) developed COVID-19 after CDI diagnosis and were excluded from the comparative analyses. **Results:** Patients with previous or concurrent COVID-19 had higher exposure to PPIs (88.2% vs. 73.3%; *p* = 0.0009), corticosteroids (70.1% vs. 26.2%; *p* < 0.0001), cephalosporins (72.9% vs. 61.6%; *p* = 0.034) and macrolides (37.5% vs. 20.3%; *p* = 0.0007). Overall antibiotic exposure was high (93.8%) and did not differ significantly between patients with previous or concurrent COVID-19 and those without COVID-19 (93.8% vs. 94.2%; *p* = 0.871). Previous or concurrent COVID-19 was not associated with increased 90-day mortality after CDI (42.4% vs. 43.6%; *p* = 0.824). **Conclusions:** Our findings suggest that a history of COVID-19 was associated with the risk factor profile, recurrence rate and the molecular epidemiology of CDI, rather than with a more severe clinical course or worse prognosis.

## 1. Introduction

*Clostridioides difficile* infection (CDI) remains a major challenge in healthcare despite advances in diagnosis, prevention, and treatment [1]. Slovakia, like many other countries, reports an annual increase in the number of CDI cases. The number of community-acquired episodes is also on the rise each year. Due to the higher incidence of CDI cases, hospital stays are prolonged, and the risk of complications and death is increasing, which results in higher costs for the healthcare system [2,3,4]. Another challenge for healthcare professionals is atypical presentations of CDI. In cases where diarrhoea and other main known symptoms of infection are absent, the period until diagnosis and appropriate treatment is longer [5,6]. The use of prognostic factors and scoring systems is increasingly being mentioned as a method for the early identification of patients at increased risk of severe and recurrent disease [7,8].

The COVID-19 pandemic has raised concerns about the impact of an increased number of critically ill patients and increased antibiotic use on the transmission of nosocomial infections and mortality [9]. There are conflicting data in available studies on the incidence of CDI during the pandemic. While few studies reported an increase in the number of CDI cases and a more severe course of the disease, others reported a stable or even decreasing trend in the incidence [10,11]. These differences may be due to local health care regulations, such as antibiotic stewardship and infection prevention measures [10,12,13,14].

Boeriu et al. reported that patients with concurrent CDI and COVID-19 had a higher burden of comorbidities, greater exposure to antibiotics and corticosteroids, and worse clinical outcomes compared with patients with CDI before the pandemic. Their analysis focused on concurrent infection and comparisons between the pre-pandemic and pandemic periods, while the role of previous or concurrent COVID-19 in CDI recurrence, drug exposure, ribotype distribution, and mortality remains unclear [15]. Although available studies from Slovakia also covered the COVID-19 pandemic period, they did not specifically evaluate the association between previous or concurrent COVID-19 infection and the course of CDI [16,17].

The local molecular characteristics of isolates may also have a significant impact on incidence and disease severity. The characteristics of different PCR ribotypes vary in terms of the presence of toxin gene profiles, antibiotic resistance, and epidemic potential. In the EUCLID study, RT176 accounted for up to 38% of all isolates in the Czech Republic, and the authors highlighted its similarity to RT027 and its potential to cause an outbreak [18]. The local significance of this ribotype has also been previously described in other studies from the Czech Republic, Poland, Croatia, and Slovakia [19,20,21,22]. Local surveillance data from Slovakia describe the predominance of fluoroquinolone-resistant RT176 and RT001, which accounted for 85% of isolates. Further studies from Eastern Slovakia have also confirmed the predominance of RT176 (61.1% and 47.6%) in isolates collected in the years 2020 and 2021 [16,17].

Therefore, the aim of our study was to evaluate whether previous or concurrent COVID-19 infection in patients with CDI was associated with differences in clinical presentation, laboratory findings, antibiotic exposure, further treatment, recurrence rates, ribotype distribution, and mortality.

## 2. Results

This study included 324 patients diagnosed with *Clostridioides difficile* infection. Previous or concurrent COVID-19 was documented in 144 patients, 172 patients had no history of COVID-19, and 8 patients developed COVID-19 after CDI diagnosis. Since the occurrence of COVID-19 following an episode of CDI could not have changed the clinical course or exposure factors, eight patients were excluded from the comparisons based on COVID-19 status. This means that the comparative analyses of previous and concurrent COVID-19 versus non-COVID-19 cases included 316 patients (144 vs. 172), while the descriptive analyses of the CDI cohort consisted of all 324 patients unless otherwise specified.

Overall, the cohort was balanced by sex, with 164 women (50.6%) and 160 men (49.4%). Their median age was 72 years, and most patients were 65 years or older (231 patients, 71.3%). The majority of cases represented hospital-acquired CDI (257 patients, 79.3%). All-cause mortality during the complete follow-up period was 58.0% (*N* = 188/324), with 52.5% (*N* = 170/324) of patients dying within one year of being diagnosed with CDI. Eighteen deaths occurred more than one year after the diagnosis of CDI. When mortality was assessed according to time from CDI diagnosis, 30-day, 60-day, and 90-day mortality were 30.9%, 40.4%, and 43.5%, respectively. The mortality rate was numerically higher among men than among women, but this difference was not statistically significant (60.6% vs. 55.5%; *p* = 0.349). The 90-day mortality rate was also not statistically different between men and women (45.6% vs. 41.5%; *p* = 0.450).

### 2.1. Clinical Characteristics According to COVID-19 Status

Patients with previous or concurrent COVID-19 were comparable to those without a history of COVID-19 regarding age, sex, and origin of CDI. The proportion of women was similar in both groups (54.2% vs. 48.8%; *p* = 0.345), as was the proportion of patients aged ≥65 years (70.1% vs. 72.7%; *p* = 0.619). Hospital-acquired CDI (HA-CDI) was slightly more frequent among patients with COVID-19, but the difference was not statistically significant (82.6% vs. 75.6%; *p* = 0.126).

The primary outcome, 90-day mortality, did not differ significantly between patients with previous or concurrent COVID-19 and those without COVID-19 (42.4% vs. 43.6%; OR 0.95, 95% CI 0.61–1.49; *p* = 0.824). Similarly, 30-day mortality (33.3% vs. 27.9%; OR 1.29, 95% CI 0.80–2.09; *p* = 0.296) and 60-day mortality (39.6% vs. 40.1%; OR 0.98, 95% CI 0.62–1.54; *p* = 0.923) were comparable between the two groups. Crude overall all-cause mortality was lower in patients with previous or concurrent COVID-19 than in those without COVID-19 (50.0% vs. 64.0%; *p* = 0.012).

The median length of hospitalization in our cohort was 17 days (IQR 12–26; 2–152 days). Patients with a history of or concurrent COVID-19 were hospitalized for a longer period, but the difference was not statistically significant (18 days, IQR 12–26 vs. 17 days, IQR 11–25; *p* = 0.525). We did not observe a statistically significant effect of the length of hospitalization on 90-day mortality, even though patients who died were hospitalized longer (18 days, IQR 12–25 vs. 17 days, IQR 11–26; *p* = 0.466). In patients with CDI recurrence, the median length of the hospital stay was 16 days (IQR 12.5–22.5) and in patients without recurrence, the median was 17 days (IQR 11–26; *p* = 0.691).

Recurrence was more common in patients with previous or concurrent COVID-19 (18.1% vs. 9.9%; OR 2.01, 95% CI 1.04–3.87; *p* = 0.035). These patients were also more often exposed to proton pump inhibitors (88.2% vs. 73.3%; OR 2.73, 95% CI 1.48–5.01; *p* = 0.0009) and corticosteroids (70.1% vs. 26.2%; OR 6.63, 95% CI 4.05–10.85; *p* < 0.0001). In contrast, the need for ICU stay (14.6% vs. 8.7%; OR 1.79, 95% CI 0.88–3.61; *p* = 0.102), a previous episode of CDI (13.9% vs. 13.4%; OR 1.05, 95% CI 0.55–1.99; *p* = 0.894) and intravenous antibiotic use during CDI (36.8% vs. 27.3%; OR 1.55, 95% CI 0.96–2.49; *p* = 0.071) did not differ significantly between the groups. In multivariable logistic regression, the association between previous or concurrent COVID-19 infection and CDI recurrence did not reach statistical significance after adjustment for age, sex, use of PPIs, corticosteroids, cephalosporins, and macrolides (aOR 1.92, 95% CI 0.92–4.03; *p* = 0.083).

#### 2.1.1. Laboratory Parameters According to COVID-19 Status

Most laboratory parameters at CDI diagnosis were comparable between the two groups. Complete white blood cell count, neutrophil count, eosinophil count, neutrophil-to-lymphocyte ratio, CRP, IL-6, and length of hospitalization were comparable between groups. The median of WBC was 12.89 × 10^9^/L (IQR 9.48–19.38) in the COVID-19 group and 13.05 × 10^9^/L (IQR 9.44–17.91) in the non-COVID-19 group (*p* = 0.810), median of CRP leve was 81.75 mg/L (IQR 26.25–128.02) and 91.16 mg/L (IQR 32.32–146.69), respectively (*p* = 0.208). IL-6 levels also did not differ significantly (39.12 ng/L, IQR 15.51–110.30 vs. 49.15 ng/L, IQR 22.21–115.40; *p* = 0.397). Lymphocyte counts were lower in patients with previous or concurrent COVID-19 than in those without COVID-19 (1.04 × 10^9^/L, IQR 0.60–1.77 vs. 1.21 × 10^9^/L, IQR 0.84–1.78; *p* = 0.045), while creatinine levels were also lower in this group (75.40 µmol/L, IQR 61.35–98.50 vs. 86.25 µmol/L, IQR 64.20–127.25; *p* = 0.046). Albumin levels were numerically higher among patients with previous or concurrent COVID-19, but this difference did not reach statistical significance (28.00 g/L, IQR 24.75–30.85 vs. 26.50 g/L, IQR 22.78–30.40; *p* = 0.052).

#### 2.1.2. Antibiotic Exposure Before CDI Diagnosis

**Figure 1:**
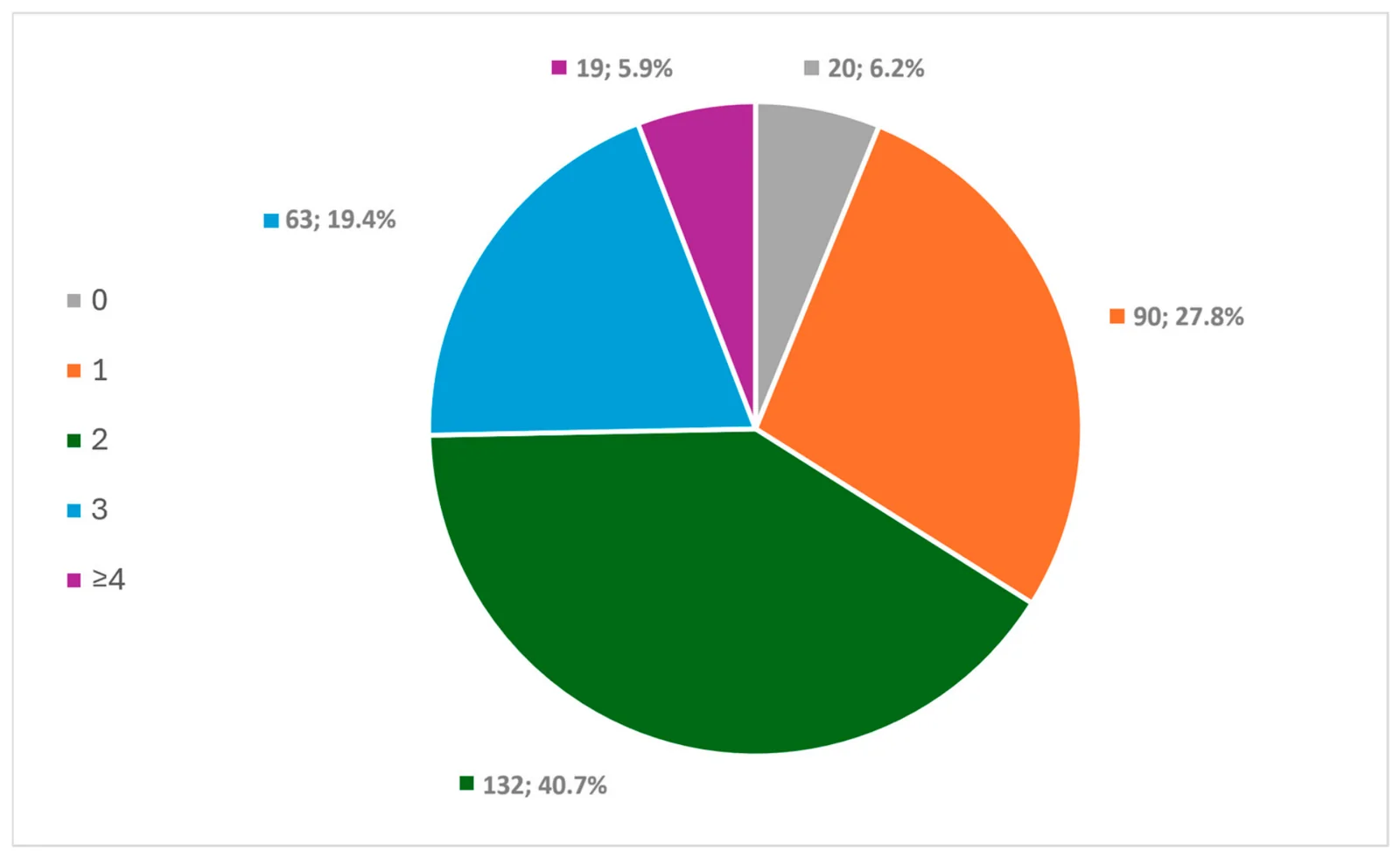
Distribution of patients with history of antibiotic use before the development of CDI according to the number of antibiotics used prior to the CDI episode. The labels 0, 1, 2, 3 and ≥4 indicate the number of antibiotic groups administered to the patients. The category ≥4 includes patients exposed to four or more antibiotic classes. The values in the graph represent the absolute number of patients and their percentage of the total sample (*N* = 324).

In the entire analysed cohort, according to the number of antibiotic classes used before CDI onset, 20 patients (6.2%) had no documented antibiotic exposure, 90 patients (27.8%) had received one antibiotic class, and 132 patients (40.7%) had taken two classes of antibiotics prior to the onset of CDI. In total, 82 patients (25.3%) had been exposed to at least three antibiotic classes before CDI diagnosis (Figure 1). Previous antibiotic exposure was prevalent in both subsequently analysed groups. Exposure to at least one antibiotic before CDI was present in 93.8% of patients with previous or concurrent COVID-19 and in 94.2% of patients without COVID-19 (OR 0.93, 95% CI 0.37–2.34; *p* = 0.871). However, the type of antibiotics differed between groups.

Patients with a history of COVID-19 infection were more often exposed to cephalosporins (72.9% vs. 61.6%; OR 1.68, 95% CI 1.04–2.71; *p* = 0.034) and macrolides (37.5% vs. 20.3%; OR 2.35, 95% CI 1.42–3.88; *p* = 0.0007). By contrast, co-trimoxazole (4.2% vs. 11.0%; OR 0.35, 95% CI 0.14–0.90; *p* = 0.024) and lincosamides (4.2% vs. 16.3%; OR 0.22, 95% CI 0.09–0.56; *p* = 0.0005) were more common in patients without COVID-19 infection. No significant differences were observed for other antibiotics.

Cephalosporins were used across all hospital departments, and there was no statistical difference between departments (*p* = 0.263). Macrolides were used primarily in the Department of Infectious Diseases and Pulmonology (*p* = 0.019). The most noticeable difference was observed with lincosamides, which were used with significantly greater frequency in the surgical departments (*p* < 0.0001). Exposure to co-trimoxazole did not show any significant differences (*p* = 0.063).

### 2.2. Characteristics of C. difficile Isolates and Ribotype Distribution

Molecular characterization was available for 314 isolates (96.9%) of the 324 patients included in the study (Table 1). Genes *tcdA* and *tcdB* were detected in 306 isolates (97.5%), and genes *cdtA* and *cdtB* for binary toxin were detected in 224 isolates (71.3%).

The *tcdA+/tcdB+/cdtA+/cdtB+* genotype was found in 224 (71.3%) isolates, the *tcdA+/tcdB+/cdtA-/cdtB-* genotype was found in 82 (26.1%) isolates, and 8 (2.5%) isolates were non-toxigenic (*tcdA-/tcdB-/cdtA-/cdtB-)*. Molecular characterization was performed subsequently for research purposes and was not used as a criterion for CDI diagnosis.

PCR ribotyping was successfully performed for 274 (87.3%) of the 314 available isolates. Ribotyping was not performed for 36 isolates (11.5%) due to technical and resource-related limitations, and it was unsuccessful for 4 isolates (1.3%). The most frequent PCR ribotype was RT176 (*N* = 172, 62.8%), followed by RT001 (*N* = 48, 17.5%) and RT027 (*N* = 19, 6.9%). Less commonly identified ribotypes were new RT (*N* = 7), RT014 (*N* = 4), RT078 (*N* = 4) and RT018 (*N* = 3). The remaining isolates were from sporadically identified ribotypes (RT005, RT010, RT017, RT020, RT176-like, RT066/2, RT011, RT013 and RT081) each represented by one or two isolates.

No significant association was found between binary toxin positivity and hospital-acquired CDI, 90-day mortality, or CDI recurrence. RT176 isolates were more often associated with previous exposure to cephalosporins (74.4% vs. 57.4%; OR 2.16, 95% CI 1.28–3.64; *p* = 0.0036) and macrolides (36.6% versus 21.8%; OR 2.08, 95% CI 1.18–3.65; *p* = 0.0105) than other isolates. Patients with RT176 had a more frequent history of COVID-19 infection (49.4% vs. 37.6%), but the difference was not statistically significant (OR 1.62, 95% CI 0.98–2.67; *p* = 0.059). We did not observe significant differences in 90-day mortality or CDI recurrence between RT176 and other isolates.

RT176 showed a specific resistance profile in antimicrobial susceptibility testing (Table 2). Its isolates were significantly more resistant to rifaximin (79.4% vs. 16.2%; OR 19.93, 95% CI 6.95–57.14; *p* < 0.0001), rifampicin (97.1% vs. 42.2%; OR 45.22, 95% CI 12.94–158.03; *p* < 0.0001) and tetracycline (33.8% vs. 8.1%; OR 5.79, 95% CI 1.61–20.90; *p* = 0.012) than all other ribotypes. In contrast, no statistically significant differences were found for susceptibility to metronidazole, vancomycin, tigecycline, doxycycline, or teicoplanin.

Most hospitalized patients with CDI originated from internal medicine departments (*N* = 129; 39.8%). These were followed by the Department of Infectious Diseases and Travel Medicine (*N* = 88; 27.2%) and the Surgery departments (*N* = 55; 17.0%). Patients with COVID-19 infection were most frequently hospitalized at the Department of Infectious Diseases and Travel Medicine and Pulmonology (Table 3). We also observed differences in mortality rates among the various departments (Table 4). However, evaluation of case incidence and mortality across individual departments is complicated by changes in the scope of care provided by some departments during the COVID-19 pandemic.

Together, Infectious Diseases and Internal Medicine departments represented approximately two-thirds of all cases (*N* = 217; 66.9%). RT176 was the predominant ribotype in most departments, particularly Internal Medicine (76/115, 66.1%), Infectious Diseases (52/79, 65.8%), and Pulmonology (10/16, 62.5%). In contrast, RT001 was dominant at the Department of Traumatology (14/23; 60.9%). In this department, nearly all patients with RT001 (13/14; 92.9%) had no history of COVID-19. RT176 was more frequent in patients with a history of COVID-19 than in those without COVID-19 (69.1% vs. 56.6%; OR 1.72, 95% CI 1.04–2.85; *p* = 0.047). On the other hand, the frequencies of RT001 (13.0% vs. 21.4%; OR 0.55, 95% CI 0.29–1.05; *p* = 0.102) and RT027 (7.3% vs. 6.9%; OR 1.07, 95% CI 0.42–2.75; *p* = 1.000) did not differ significantly between the groups.

### 2.3. CDI Treatment

Treatment was mainly based on regimens containing vancomycin. Vancomycin was overall used in 286 patients (88.3%). Vancomycin monotherapy was the most common regimen, administered to 205 patients (63.3%). Metronidazole was used in 85 patients (26.2%), mostly in combination with vancomycin. Fidaxomicin was administered to 37 patients (11.4%), including 20 patients treated only with fidaxomicin. Patients treated with fidaxomicin had lower albumin levels (25.35 vs. 27.65 g/L; *p* = 0.038). Rifaximin and faecal microbiota transplantation were used in 12 (3.7%) and 6 (1.9%) patients.

Treatment distribution did not differ significantly between patients with a history of COVID-19 infection. The use of vancomycin was almost identical between groups (88.2% vs. 88.4%; OR 0.98, 95% CI 0.49–1.96; *p* = 0.961). Metronidazole was numerically less frequent in the COVID-19 group (22.9% vs. 30.2%; OR 0.69, 95% CI 0.41–1.14; *p* = 0.144), whereas fidaxomicin was numerically more frequent (14.6% vs. 8.7%; OR 1.79, 95% CI 0.88–3.61; *p* = 0.102), but neither difference was statistically significant.

### 2.4. All-Cause and 90-Day Mortality

All-cause mortality was 58.0%. Patients who died were significantly older, with a median age of 75.0 years compared with 65.5 years among survivors (*p* < 0.0001). Patients aged ≥65 years were also numerically more frequent among those who died (82.4% vs. 55.9%; OR 3.71, 95% CI 2.23–6.15; *p* < 0.0001). Mortality differed significantly depending on the year of CDI diagnosis, with the highest rate in 2020 and the lowest in 2021 (71.0% vs. 50.3%; overall *p* = 0.0082). Within each year, no significant differences were observed in the 30-day or 90-day mortality rates.

Mortality was associated with worse laboratory findings. Deceased patients had higher complete WBC counts (14.67 × 10^9^/L, IQR 9.62–20.78 vs. 11.72 × 10^9^/L, IQR 9.09–16.28; *p* = 0.0059), neutrophil counts (11.91 × 10^9^/L, IQR 6.73–17.52 vs. 8.78 × 10^9^/L, IQR 5.39–12.89; *p* = 0.0009), NLR values (11.92, IQR 5.44–22.15 vs. 6.82, IQR 3.71–12.59; *p* < 0.0001), CRP levels (95.57 mg/L, IQR 31.57–152.79 vs. 66.71 mg/L, IQR 28.36–118.04; *p* = 0.022) and IL-6 levels (67.59 ng/L, IQR 24.91–144.55 vs. 33.99 ng/L, IQR 11.18–61.18; *p* = 0.0002). In contrast, deceased patients had significantly lower lymphocyte counts (1.04 × 10^9^/L, IQR 0.60–1.66 vs. 1.24 × 10^9^/L, IQR 0.94–1.85; *p* = 0.0029), eosinophil counts (0.02 × 10^9^/L, IQR 0.01–0.11 vs. 0.08 × 10^9^/L, IQR 0.01–0.16; *p* = 0.0014) and albumin levels (25.60 g/L, IQR 22.63–29.20 vs. 28.85 g/L, IQR 26.12–33.08; *p* < 0.0001).

Follow-up for all-cause mortality was monitored until December 2025. The median follow-up time from CDI diagnosis to vital status assessment was 1645 days (IQR 1424–1767) in patients with previous or concurrent COVID-19 and 1721 days (IQR 1486–2098) in patients without COVID-19. Although crude overall all-cause mortality was lower in the COVID-19 group (50.0% vs. 64.0%; *p* = 0.012), this association did not reach statistical significance in time-to-event analysis. In Cox proportional hazards regression, previous or concurrent COVID-19 was not significantly associated with all-cause mortality (HR 0.78, 95% CI 0.58–1.06; *p* = 0.110).

Death within 90 days after CDI diagnosis occurred in 141 patients (43.5%). Patients who died within 90 days were significantly older, with a median age of 76.0 years compared with 68.0 years (*p* < 0.0001). The proportion of patients ≥65 years was also higher among patients who died within 90 days of CDI (85.1% vs. 60.7%; OR 3.71, 95% CI 2.14–6.42; *p* < 0.0001).

Previous or concurrent COVID-19 was not associated with increased 90-day mortality (42.4% vs. 43.6%; OR 0.95, 95% CI 0.61–1.49; *p* = 0.824). Patients who died within the 90-day period had higher WBC counts (15.60 vs. 11.72 × 10^9^/L; *p* < 0.0001), neutrophil counts (13.68 vs. 8.41 × 10^9^/L; *p* < 0.0001), NLR (14.87 vs. 6.48; *p* < 0.0001), creatinine levels (94.80 vs. 75.50 µmol/L; *p* = 0.0004), CRP levels (107.66 vs. 64.75 mg/L; *p* < 0.0001) and IL-6 levels (85.64 vs. 31.45 ng/L; *p* < 0.0001). They also had lower lymphocyte counts (0.93 vs. 1.22 × 10^9^/L; *p* = 0.0002), eosinophil counts (0.01 vs. 0.08 × 10^9^/L; *p* < 0.0001), and albumin levels (25.05 vs. 28.90 g/L; *p* < 0.0001). In multivariable logistic regression adjusted for age, sex, NLR, albumin, creatinine, and IL-6, previous or concurrent COVID-19 was not independently associated with 90-day mortality (aOR 1.21, 95% CI 0.50–2.90; *p* = 0.668).

## 3. Discussion

Our results show that, during the pandemic, COVID-19 was associated with differences in clinical and epidemiological characteristics among patients with CDI. The severity of the disease did not worsen in patients with COVID-19, despite the identification of a slightly different set of risk factors, including use of PPIs, corticosteroids, cephalosporins, and macrolides. The incidence of recurrences and RT176-associated local outbreaks was also higher, but without a significant increase in patient mortality. Our results suggest that the ongoing pandemic primarily affected the circumstances leading to the development of CDI, while the prognosis of the patient was not further significantly affected.

The relationship between *C. difficile* and COVID-19 is not uniformly described in studies. Spigaglia describes the relationship between COVID-19 and CDI as a highly complex problem [23]. While stricter hygiene measures have led to a decline or stabilization in CDI incidence in some countries, hospital overcrowding, the overuse of broad-spectrum antibiotics, and a growing group of high-risk patients who have recovered from COVID-19 have together created a new risk profile for the development of CDI [12,23,24,25]. There were no significant differences in the demographic characteristics between patients in the two study groups. We observed similar age groups, sex distribution, and comparable incidence of hospital-acquired infections. Similar observations are reported by Vázquez-Cuesta et al., who also described comparable demographic characteristics of the patients [12]. In studies focusing on CDI without the presence of COVID-19, a higher incidence among women (55–60%) is reported, with a more apparent difference in community-acquired cases [1,26].

The duration of hospital stay of patients is affected not only by the severity of their illness, but also by non-patient factors such as hospital occupancy rates, admission criteria, and the organization of healthcare during the pandemic. Rees et al., in their systematic review, describe significant variability in the length of hospitalization across countries and studies (4–53 days within China; 4–21 days outside of China). This finding emphasizes the need to assess this parameter at the local level [27]. In our cohort, the median length of hospitalization was 17 days. Patients with previous or current COVID-19 were hospitalized only numerically longer than patients without COVID-19 (18 days, IQR 12–26 vs. 17 days, IQR 11–25; *p* = 0.525). Similar results were found by Markovic-Denic et al., who did not observe a significant difference in the length of hospital stay of HA-CDI patients between the COVID-19 group and the non-COVID-19 group [13]. In contrast, other studies reported that coinfection was associated with a longer hospitalization (13.57 ± 8.34 days vs. 9.21 ± 9.02 days) and more severe outcomes [28].

Among the most commonly mentioned modifiable risk factors for CDI is the use of drugs, especially antibiotics and PPIs [1,26,29]. The non-antibiotics identified as risk factors in a population-based case-control study from Sweden included antidiarrheals, corticosteroids, PPIs, nervous system drugs, constipation medications, histamine H2-receptor antagonists, antidepressants, and beta blockers [29]. According to a meta-analysis by Langford et al. at the start of the pandemic, almost three-quarters of patients with COVID-19 were receiving antibiotics. With these findings, they highlighted the risk that increased antibiotic use could contribute to patient harm and to global antibiotic resistance [30]. Similar findings related to high exposure to drugs associated with a high risk of *Clostridioides difficile* were also observed in our study sample. Previous antibiotic exposure was documented in 93.8% (*N* = 304/324), PPI exposure in 79.6% (*N* = 258/324), and corticosteroid exposure in 46.9% (*N* = 152/324) of patients. Use of these medications was also associated with an increased risk of *C. difficile* colonization [31]. It is important to mention that the concurrent use of antibiotics and PPIs has been identified as one of the most significant risk combinations for both CDI and recurrent CDI [32].

In our cohort, we observed that antibiotic use prior to the development of CDI occurred with approximately the same frequency in patients without a history of COVID-19 and in those with a history of COVID-19 (93.8% vs. 94.2%; *p* = 0.871). However, cephalosporins and macrolides were used more frequently in patients with ongoing or previous COVID-19. Our findings reflect the empirical treatment used for secondary bacterial pneumonia in our departments during COVID-19. This group of patients was also more frequently exposed to PPIs and corticosteroids for the same reason. The distribution of patients across departments likely reflected the temporary repurposing of departments for care of COVID-19 patients. We therefore did not make further comparisons between departments.

Molecular characteristics confirmed the predominant presence of toxigenic *Clostridioides difficile* isolates (*N* = 306; 97.5%) and, at the same time, a high proportion of strains positive for the binary toxin (*N* = 224; 71.3%), which was likely associated with the highly prevalent RT176. This ribotype was dominant across the entire specimen set, with 172 of the 274 successfully ribotyped isolates (62.8%) belonging to this ribotype. The higher proportion of RT176 among patients with a history of COVID-19 may be related to local hospital epidemiology, the antimicrobial therapy used, and the department reorganization during the pandemic.

Whole-genome sequencing of RT176 revealed significant similarity to the hypervirulent RT027, which is frequent in Western European countries. Therefore, this ribotype is also referred to as 027-like [20,33,34]. For several years, the dominance of RT176 reflected the epidemiological trend in Central Europe, particularly in Slovakia and the Czech Republic [16,17,20,21,22,33,34,35]. According to subsequent data, the situation in the Czech Republic changed, with RT001 becoming dominant [36]. RT001 was the second most common ribotype in our study sample and was also the second most common according to further studies from Slovakia [17,21]. Similar findings were also reported in Greece, where the distribution of the most prevalent *C. difficile* ribotypes did not change during the COVID-19 pandemic and ribotypes RT181, RT126, and RT017 were still the most common [37].

The resistance profile of RT176 in our study, particularly higher resistance or intermediate susceptibility to rifaximin, rifampicin, and tetracycline, was consistent with previous regional data [16,17]. High rates of resistance to moxifloxacin have been reported in the dominant isolates RT176 and RT001 [21]. These results emphasize the role of selective pressure from antimicrobials in the hospital environment that may contribute to the persistence and spread of epidemic ribotypes of *C. difficile*.

Recurrence of CDI was more common in patients with a previous or concurrent COVID-19 infection than in patients without COVID-19 (18.1% vs. 9.9%; *p* = 0.035). This finding may be explained by the combination of risk factors associated with recurrence in the COVID-19 group, including more frequent exposure to PPIs, corticosteroids, cephalosporins, and macrolides. However, after adjustment for age, sex, PPI exposure, corticosteroid exposure, cephalosporin exposure, and macrolide exposure, the association between previous or concurrent COVID-19 and CDI recurrence decreased and was not statistically significant. The higher recurrence rate in patients with previous or concurrent COVID-19 may partly reflect differences in drug exposure and patient characteristics rather than a direct effect of COVID-19. Therefore, residual confounding cannot be excluded. Several studies suggest the same trend, with the highest risk of recurrence observed in patients receiving antibiotics, PPIs, patients of older age, and those with prior hospitalization [8,38]. In contrast to our findings, in which prior or concurrent COVID-19 was not associated with increased 90-day mortality but was associated with a significantly higher recurrence rate, Vázquez-Cuesta et al. reported significantly higher in-hospital mortality among patients with HA-CDI and COVID-19 and a numerical, but not statistically significant, increase in the CDI recurrence rate [12]. In a systematic review from 2000 to 2010, the incidence of recurrent CDI ranged from 1% in France to 36% in Ireland, but the definitions of recurrence were different depending on the study [39]. Finn et al. reported in their systematic review that recurrent CDI occurred in approximately 10–20% of patients following an initial episode of CDI. The median recurrence rate in the included studies was 17.0%, but the range was very wide, from 0% to 64%, again depending on the patient population and the definition used [38].

The study, which analysed deaths associated with CDI from 63 countries, including 30 European countries, between 2001 and 2023, found that the most significant decreasing trends were observed in the United States and in countries with a high sociodemographic index [40]. We also observed a difference in mortality rates from year to year among our patients, with mortality rates of 71.0% (*N* = 49/69) in 2020, 50.3% (*N* = 84/167) in 2021, and 62.5% (*N* = 55/88) in 2022. In our cohort, the 30-day mortality rate was 30.9% overall, with mortality rates being comparable across the individual years of the study, at 26.1% in 2020, 32.3% in 2021, and 31.8% in 2022. Similarly, 90-day mortality did not differ significantly across the years, reaching 43.5% in 2020, 41.3% in 2021, and 47.7% in 2022. In a systematic review from 14 countries, the 30-day mortality rate for CDI varied from as low as 2% in France to as high as 42% in the UK [39,41]. Differences in the data were also observed in a retrospective case-control study examining 90-day mortality among hospitalized patients from 2010 to 2019 in seven European countries, with mortality reaching 66.5% [42]. In contrast, a significantly lower rate was reported in a study of patients from Denmark, where it was 28% [43]. Different mortality rates across countries may be partly related to differences in the local distribution of ribotypes. European studies have shown differences in circulating *C. difficile* ribotypes, and ribotype-specific differences in mortality have been observed, particularly for RT027 and RT176 [18,44,45,46,47]. Therefore, when comparing CDI outcomes across countries, not only patient factors but also local epidemiology should be taken into consideration.

Differences in laboratory parameters were more pronounced when patients were analysed according to mortality. Patients who died had higher white blood cell counts, higher neutrophil counts, higher NLR, higher levels of creatinine, CRP, and IL-6. In contrast, they had lower lymphocyte counts, eosinophil counts, and albumin levels. A similar pattern was observed for 90-day mortality, with the most pronounced differences seen in inflammatory markers and albumin levels. These findings suggest that mortality after CDI was more closely associated with the immune and inflammatory response of the host than with COVID-19 status, since laboratory parameters did not differ significantly between patients who had overcome COVID-19 and those who were currently infected. In addition to the usual laboratory parameters commonly used to evaluate the severity of CDI, including WBC, CRP, creatinine, and albumin, our findings also highlight the prognostic significance of other inflammatory and immune markers [1,48,49,50]. Wang et al. have identified low peripheral eosinophil counts as a predictor of 30-day all-cause mortality in CDI, with eosinopenia being strongly associated with death in the validation cohort (adjusted OR 2.49, 95% CI 1.77–3.50) [51]. Scarlata et al. describe neutrophils and the NLR as useful biomarkers for predicting mortality in patients with CDI [52]. Higher NLR values and low lymphocyte counts are also associated with disease severity and all-cause mortality in hospitalized patients with COVID-19 [53,54].

The treatment regimen did not differ significantly based on the presence of COVID-19. ESCMID guidelines changed during the study period [55,56]. Therefore, treatment choices in our cohort should be interpreted in the context of changing guidelines, local availability, costs, and clinical decisions. Vancomycin was the predominant treatment option used in our cohort. Until 2021, it was used as first-line therapy, when newer ESCMID and IDSA/SHEA guidelines were published, recommending the use of fidaxomicin [56,57]. Over the study period, the use of fidaxomicin was preferred in patients considered to be at higher risk of severe course, recurrence, and death. This reflects the local choice of treatment and should be considered when interpreting the distribution of antibiotics. During the study period, given its cost, fidaxomicin was used in our hospital only for the patients with the highest risk of recurrence. This approach is supported by the findings of a systematic review and meta-analysis published by Zhao et al., which showed that while fidaxomicin was superior to vancomycin regarding recurrence rate and mortality, vancomycin was most effective in treating severe CDI [58].

This study has several limitations. Its retrospective and single-centre design may limit the accuracy of the data and the universal validity of the findings. Furthermore, the local prevalence of the RT176 should be taken into consideration when interpreting the molecular epidemiology results. It was not possible to clearly separate COVID-19 status from COVID-19-related treatment exposures, patient characteristics, and hospital organisational changes during the pandemic; therefore, residual confounding cannot be excluded. In addition, PCR ribotyping data were not available for all isolates. Finally, all-cause mortality may have reflected age, comorbidities and underlying conditions and should not be interpreted as mortality associated with CDI.

## 4. Material and Methods

### 4.1. Study Population and Clinical Data Collection

An observational retrospective study was conducted involving 324 adult patients hospitalized at the Louis Pasteur University Hospital in Kosice, Slovakia, between 2020 and 2022. All subjects included presented with clinical symptoms of *Clostridioides difficile* infection (CDI) and were diagnosed using a standardized algorithmic approach combining clinical symptoms (diarrhoea), GDH and toxin A/B enzyme immunoassay, and molecular confirmation of toxigenicity. Clinical records were reviewed to extract data on demographics, comorbidities, and outcomes, including mortality and CDI recurrence. Cases were classified as hospital-acquired CDI or community-acquired CDI according to the available clinical and epidemiological data. Any episode that occurred on day 3 or later following admission to hospital or with onset in the community within 4 weeks after discharge from a healthcare facility was defined as hospital-acquired [59]. Previous or concurrent COVID-19 was defined as documented infection occurring within 3 months before CDI diagnosis or at the time of CDI diagnosis. The interval between COVID-19 and CDI diagnosis was calculated from the date of the last positive test to the date of CDI diagnosis. Mortality was assessed as all-cause mortality; deaths occurring within 30, 60, and 90 days after CDI diagnosis were analysed separately. CDI recurrence was defined as a documented recurrent episode occurring within 8 weeks after the previous CDI episode [59].

A significant focus was placed on the history of previous or concurrent COVID-19. Furthermore, exposure to predisposing medications, such as proton pump inhibitors (PPIs), corticosteroids, and various antibiotic classes (e.g., cephalosporins, macrolides, and lincosamides), was documented for each patient. Laboratory parameters were analysed using values obtained closest to the day of CDI diagnosis, within a predefined window from two days before to two days after diagnosis. Missing laboratory values were excluded from analyses of the respective parameters.

### 4.2. Laboratory and Microbiological Procedures

Initial screening of stool samples was performed using rapid immunochromatographic assays for GDH and toxins A/B. All stool samples evaluated as GDH-positive in the phenotypic rapid test, regardless of whether they were toxin A/B-positive or toxin A/B-negative, were subjected to anaerobic cultivation. Bacterial isolation was achieved via anaerobic cultivation on selective Brazier’s agar following alcohol pretreatment to facilitate spore selection. Species identification was subsequently verified using matrix-assisted laser desorption/ionization time-of-flight (MALDI-TOF) mass spectrometry (Bruker Daltonics, Billerica, MA, USA). To ensure a comprehensive analysis, clinical laboratory parameters were recorded at the time of CDI diagnosis, including white blood cell (WBC) count, neutrophil and lymphocyte counts, neutrophil-to-lymphocyte ratio (NLR), C-reactive protein (CRP), interleukin-6 (IL-6), creatinine and albumin levels.

### 4.3. Molecular Characterization and Toxin Gene Detection

Molecular characterization was successfully performed for 314 isolates (96.9%) of the study cohort. Bacterial genomic DNA was isolated using the GRS Genomic DNA kit according to the manufacturer’s instructions and stored at −20 °C until further processing. The detection of the pathogenicity locus genes was carried out using a single multiplex PCR assay as described by Persson et al. [60]. This specific protocol allowed for the simultaneous amplification of the toxin A (*tcdA*) and toxin B (*tcdB*) genes, as well as the binary toxin genes (*cdtA* and *cdtB*). To ensure the accuracy of *C. difficile* identification, the 16S rDNA sequence was included as an internal control in every reaction. The resulting PCR products were separated via agarose gel electrophoresis and analysed using Bio-Rad Image Lab Software (BIO-RAD, Hercules, CA, USA).

### 4.4. PCR Ribotyping

To determine the epidemiological distribution of strains, PCR ribotyping was performed on 274 isolates using capillary electrophoresis. This method targeted the 16S–23S rRNA intergenic spacer region. The resulting fluorescently labelled fragments were separated on an automated genetic analyser ABI3130 (Thermo Fisher Scientific, St. Louis, MO, USA) and the profiles were categorized by comparison with the international Webribo database [61].

### 4.5. Antimicrobial Susceptibility Testing (AST)

The susceptibility of the C. difficile isolates was evaluated against a panel of antimicrobials: vancomycin, metronidazole, rifampicin, rifaximin, tigecycline, doxycycline, tetracycline and teicoplanin. Minimum inhibitory concentrations (MICs) were determined using the automated BACMED 6iG2 system (Bacmed, Litomyšl, Czech Republic) based on broth microdilution.

Susceptibility results were interpreted according to the guidelines and versions applicable during the study period (2020–2022), specifically the European Committee on Antimicrobial Susceptibility Testing (EUCAST clinical breakpoints v12.0, 2022) and the Clinical and Laboratory Standards Institute (CLSI M11, 9th edition) guidelines [62,63]. For vancomycin, metronidazole, and teicoplanin, resistance was defined according to EUCAST clinical breakpoints (MIC > 2 mg/L). For tetracycline and doxycycline, CLSI breakpoints for anaerobic bacteria were applied (resistance defined as MIC ≥ 16 mg/L). For agents without established clinical breakpoints, epidemiological cut-off values (ECOFFs) or well-established literature-based resistance thresholds were utilized: EUCAST ECOFF of >0.25 mg/L for tigecycline and MIC > 4 mg/L and >16 mg/L for rifampicin and rifaximin, respectively [16]. 

### 4.6. Statistical Analysis

Statistical analyses were performed using IBM SPSS Statistics 25 and Python version 3.12. Categorical variables are presented as numbers and percentages, and continuous variables as medians with interquartile ranges (IQRs). Continuous variables were compared using the Mann–Whitney U test or Kruskal–Wallis test, and categorical variables using the χ^2^ test or Fisher’s exact test.

The primary outcome was 90-day all-cause mortality, and CDI recurrence was considered a secondary outcome. Effect estimates are reported as odds ratios (ORs), adjusted odds ratios (aORs), or hazard ratios (HRs), with 95% confidence intervals (CIs). Multivariable logistic regression was used for 90-day all-cause mortality and CDI recurrence. The 90-day mortality model was adjusted for age, sex, NLR, albumin, creatinine, and IL-6, while the recurrence model was adjusted for age, sex, PPI exposure, corticosteroid exposure, cephalosporin exposure, and macrolide exposure. All other comparisons were interpreted descriptively and with caution, as no formal adjustment for multiple testing was applied. A *p*-value < 0.05 was considered statistically significant.

## 5. Conclusions

In conclusion, previous or concurrent COVID-19 was associated with differences in medication use and microbiological characteristics in CDI patients, but it was not independently associated with increased 90-day all-cause mortality. Patients with previous or concurrent COVID-19 were more frequently exposed to PPIs, corticosteroids, cephalosporins, and macrolides. RT176 was the most prevalent PCR ribotype in this group. Mortality was primarily linked to advanced age and laboratory markers of disease severity. These findings should be interpreted with caution, as residual confounding related to drug exposure, patient characteristics, and hospital organisational changes during the pandemic cannot be excluded.

## Figures and Tables

**Table 1:** Overview of toxin genes and toxin gene profiles.

Molecular Characteristics	*N*	%
*tcdA* positive	306	97.5 *
*tcdA* negative	8	2.5 *
*tcdB* positive	306	97.5 *
*tcdB* negative	8	2.5 *
*cdtA* and *cdtB* positive	224	71.3 *
*cdtA* and *cdtB* negative	90	28.7 *
*tcdA+/tcdB+/cdtA+/cdtB+*	224	71.3 *
*tcdA+/tcdB+/cdtA−/cdtB−*	82	26.1 *
*tcdA−/tcdB−/cdtA−/cdtB−*	8	2.5 *

* Percentage calculated from isolates with available molecular characterization (*N* = 314).

**Table 2:** Comparison of antibiotic resistance profiles in RT176 and other ribotypes.

Antibiotic	RT176 *n*/*N* (%)	Other Ribotypes *n*/*N* (%)	*p*-Value
Rifaximin	54/68 (79.4)	6/37 (16.2)	<0.0001
Rifampicin	99/102 (97.1)	27/64 (42.2)	<0.0001
Tetracycline	23/68 (33.8)	3/37 (8.1)	0.012
Vancomycin	14/170 (8.2)	3/67 (4.5)	0.212
Metronidazole	8/170 (4.7)	2/67 (3.0)	0.654
Tigecycline	2/170 (1.2)	1/33 (3.0)	0.601
Doxycycline	5/170 (2.9)	1/34 (2.9)	0.569
Teicoplanin	1/102 (1.0)	0/42 (0.0)	0.889

**Table 3:** Distribution of CDI cases by department and COVID-19 status.

Department	Previous/Concurrent COVID-19	No COVID-19	COVID-19 After CDI
Internal medicine	45 (34.9%)	79 (61.2%)	5 (3.9%)
Infectious Diseases	55 (62.5%)	32 (36.4%)	1 (1.1%)
Surgery	16 (29.1%)	38 (69.1%)	1 (1.8%)
Pulmonology	14 (70.0%)	5 (25.0%)	1 (5.0%)
Neurology	5 (41.7%)	7 (58.3%)	0
Anaesthesiology	1 (20.0%)	4 (80.0%)	0
Other departments < 5	8 (53.3%)	7 (46.7%)	0
Total	144 (44.4%)	172 (53.1%)	8 (2.5%)

**Table 4:** Overall and 90-day mortality according to department.

Department	*n*	All-Cause Mortality	90-Day Mortality
Internal medicine	129	85 (65.9%)	64 (49.6%)
Infectious Diseases	88	37 (42.0%)	29 (33.0%)
Surgery	55	33 (60.0%)	21 (38.2%)
Pulmonology	20	15 (75.0%)	14 (70.0%)
Neurology	12	8 (66.7%)	6 (50.0%)
Anaesthesiology	5	5 (100.0%)	4 (80.0%)
Other departments < 5	15	5 (33.3%)	3 (20.0%)
Total	324	188 (58.0%)	141 (43.5%)

## Data Availability

No new data were created or analyzed in this study. Data sharing is not applicable to this article.

## Abbreviations

The following abbreviations are used in this manuscript:

CDI	*Clostridioides difficile* Infection
CRP	C-reactive Protein
ESCMID	European Society of Clinical Microbiology and Infectious Diseases
HA-CDI	Hospital-Acquired *Clostridioides difficile* Infection
ICU	Intensive Care Unit
IDSA	Infectious Diseases Society of America
IL-6	Interleukin 6
IQR	Interquartile Range
NLR	Neutrophil-to-Lymphocyte Ratio
PCR	Polymerase Chain Reaction
PPI; PPIs	Proton Pump Inhibitor; Proton Pump Inhibitors
RT	Ribotype
SHEA	Society for Healthcare Epidemiology of America
WBC	White Blood Cells (Leukocytes)
